# One-month DAPT after biodegradable-polymer everolimus-eluting stent implantation in patients at high-bleeding risk: an individual patient data pooled analysis of the SENIOR and POEM trials

**DOI:** 10.1093/ehjopen/oeae068

**Published:** 2024-08-06

**Authors:** Carlo A Pivato, Giulio Stefanini, Daniele Giacoppo, Georgios Sideris, Luca Testa, Dragica Paunovic, Carlo Briguori, Ciro Indolfi, Bernhard Reimers, Peter Sinnaeve, Olivier Varenne

**Affiliations:** Department of Biomedical Sciences, Humanitas University, Via Rita Levi Montalcini 4, 20072 Pieve Emanuele, Milan, Italy; IRCCS Humanitas Research Hospital, via Manzoni 56, 20089 Rozzano, Milan, Italy; Department of Biomedical Sciences, Humanitas University, Via Rita Levi Montalcini 4, 20072 Pieve Emanuele, Milan, Italy; IRCCS Humanitas Research Hospital, via Manzoni 56, 20089 Rozzano, Milan, Italy; Policlinico ‘Rodolico-San Marco’, Department of General Surgery and Medical-Surgical Specialties, University of Catania, Catania, Italy; Paris Cardiovascular Research Center, Université Paris Cité, Paris, France; European Georges Pompidou Hospital, APHP, Paris, France; Department of Cardiology, IRCCS Policlinico San Donato, Milan, Italy; Board of Directors, European Cardiovascular Research Centre (CERC), Massy, France; Interventional Cardiology Unit, Mediterranea Cardiocentro, Naples, Italy; Department of Medical and Surgical Sciences, ‘Magna Graecia’ University, Catanzaro, Italy; IRCCS Humanitas Research Hospital, via Manzoni 56, 20089 Rozzano, Milan, Italy; Department of Cardiovascular Medicine, University Hospitals Leuven, Leuven, Belgium; Cochin Hospital, Hôpitaux Universitaire Paris Centre, Assistance Publique-Hôpitaux de Paris, Paris, France

**Keywords:** Biodegradable polymer, Everolimus, Coronary artery disease, High bleeding risk, Percutaneous coronary intervention, Short DAPT

## Abstract

**Aims:**

Dual antiplatelet therapy (DAPT) can be shortened up to 1 month in high-bleeding risk (HBR) patients receiving a contemporary biodegradable-polymer sirolimus-eluting stent. We aimed to summarize the evidence on a similar DAPT regimen after biodegradable-polymer everolimus-eluting stent (EES) implantation in patients at HBR.

**Methods and results:**

We pooled the individual participant data from the available trials evaluating this strategy, namely, the SENIOR and the POEM trials. Inclusion criteria were ≥1 biodegradable-polymer EES implantation and ≤1-month duration of DAPT. The primary endpoint was the 1-year composite of cardiovascular death, myocardial infarction, or stroke. Major bleeding was defined as Bleeding Academic Research Consortium (BARC) type 3–5 bleeding. Landmark analyses were performed at 1 month, the time point for intended DAPT interruption. We included 766 participants (age 77.5 ± 8.2 years, women 31.9%), 323 from the SENIOR and 443 from the POEM trial. The primary endpoint occurred in 45 participants (6.0%; 95% confidence interval [CI], 4.3–7.7%) through 1 year of follow-up, with 21 (2.8%; 95% CI, 1.6–3.9%) events during the first month and 24 (3.4%; 95% CI, 2.0–4.7%) thereafter. The incidences of cardiovascular death, myocardial infarction, and stroke were 2.2% (95% CI, 0.36–2.50%), 3.1% (95% CI, 1.8–4.3%), and 1.2% (95% CI, 0.4–2.0%), respectively. BARC type 3–5 bleeding ocuurred in 1.1% (95% CI, 0.3–1.8%) at 1 month and 2.9% (95% CI, 1.6–4.1%) at 1 year.

**Conclusion:**

HBR patients receiving biodegradable-polymer EES had few ischemic and bleeding events when given 1 month of DAPT. One-month DAPT after biodegradable-polymer EES implantation seems safe in patients at HBR.

## Graphical Abstract

**Graphical Abstract:**
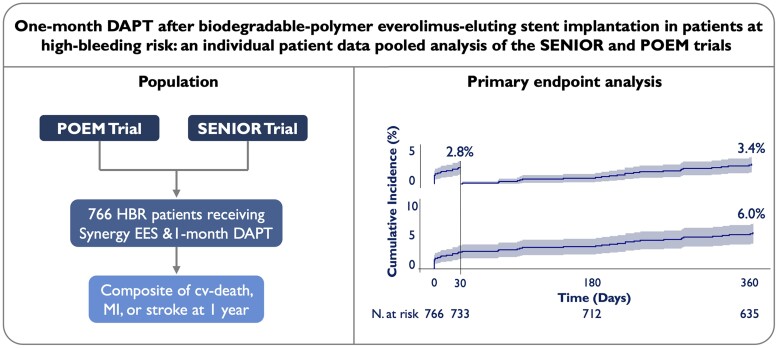

## Introduction

Dual antiplatelet therapy (DAPT) after percutaneous coronary intervention (PCI) reduces the occurrence of ischemic events at the cost of a higher risk of bleeding. However, bleeding events during DAPT can have a prognostic impact comparable to that associated with ischemic events.^1,2^ These findings are remarkable considering that up to 40% of patients undergoing PCI are deemed to be at high bleeding risk (HBR).^3^ Antiplatelet strategies balancing the antithrombotic requirements with the associated risk of bleeding can improve the outcome of these patients.^4^

Several randomized trials have shown that contemporary drug-eluting stents (DES) allow short DAPT as device-related ischemic events were generally comparable with more prolonged regimens.^5^ Of note, short DAPT was associated with reduced bleeding in some of these trials.^5^ Recently, the MASTER-DAPT trial demonstrated that DAPT could be shortened up to 1 month in HBR patients receiving a contemporary biodegradable-polymer sirolimus-eluting stent (SES, Ultimaster, Terumo, Japan), allowing fewer bleeding and similar thrombotic events compared with standard DAPT duration.^6^ It is unclear if these results can be generalized to other contemporary DES.

The Synergy everolimus-eluting stent (EES) (Boston Scientific Corporation, USA) is a thin-strut (74–81 μm) platinum–chromium metal alloy platform coated with an ultrathin (4 μm) biodegradable poly(Dl-lactide-co-glycolide) polymer on the abluminal side.^7^ These features may allow rapid stent reendothelialization and low thrombogenicity, both key characteristics to shorten DAPT.^8,9^ In the SENIOR trial, the Synergy EES was proven superior in terms of a composite of major adverse cardiac and cerebrovascular events (MACCE) to thin-strut bare-metal stents (BMS) in elderly patients.^10^ In this trial, the intended DAPT duration was 1 month for participants with chronic and 6 months for those with acute coronary syndrome.^10^ In the POEM trial, 1-month DAPT was associated with favourable ischemic and bleeding event rates in patients at HBR undergoing PCI with the Synergy EES.^11–13^ However, the study was underpowered due to a lower-than-anticipated number of enrolled participants.

We aimed to summarize the available evidence on 1-month DAPT after Synergy EES stent implantation in participants at HBR by pooling together the trials evaluating this strategy.

## Methods

The data underlying this article will be shared upon reasonable request to the corresponding author.

### Study design and population

We pooled individual participant data from the SENIOR and POEM trials because they evaluated a strategy of 1-month DAPT following PCI with the implantation of the Synergy EES. Supplementary material online, *Table S1* summarizes and compares the characteristics of both trials, including the main inclusion and exclusion criteria. The institutional review board of each centre approved the protocols of corresponding studies, which were conducted following the ethical principles of the Declaration of Helsinki. Participants were required to provide written informed consent before participating in each study.

### Data collection and harmonization

The Cardiovascular European Research Center (Massy, France), an independent research organization, was the data coordinating centre. Individual participant data included baseline clinical characteristics and demographics, procedural features, relevant discharge information, and clinical outcomes at follow-up. These data were anonymized, extracted, transferred in preformatted electronic spreadsheets, and finally merged into a core study dataset. Inclusion criteria were 1) the implantation of at least one Synergy EES and 2) an intended DAPT duration equal to or less than 1 month. The implantation of at least one BMS represented the sole exclusion criterion.

### Study endpoints

The primary endpoint was MACCE, a composite of cardiovascular death, myocardial infarction, or stroke at 12 months. Secondary endpoints at 12 months included all-cause death, cardiovascular death, non-cardiovascular death, myocardial infarction, stent thrombosis, target-vessel revascularization, target-lesion revascularization, major/severe (i.e. type 3–5) bleeding according to Bleeding Academic Research Consortium (BARC) criteria, net adverse cardiac and cerebrovascular event (NACCE), a composite of cardiovascular death, myocardial infarction, stroke, or BARC type 3–5 bleeding. Details on clinical event adjudication and study definitions used in each trial are provided in Supplementary material online, *Table S1*. The outcome definitions were consistent across the included studies: myocardial infarction was defined according to the third Universal Definition of Myocardial Infarction,^14^ stent thrombosis according to the Academic Research Consortium (ARC) definitions,^15^ and bleeding according to the BARC definitions.^16^

### Statistical analysis

Categorical data are reported as counts and proportions. Continuous variables are reported as mean ± standard deviation or median (interquartile range), as appropriate. The cumulative distribution of outcomes from the index PCI to 1-year follow-up was computed using the Kaplan–Meier method. Landmark analyses were performed at 1 month, which was the time point for intended DAPT interruption. The associations between main outcomes and baseline conditions were assessed by multivariable Cox proportional hazards regression model employed on datasets (n = 5) generated by chained equation multiple imputation. The results were combined using Rubin's rules and expressed as hazard ratios (HRs) and 95% confidence intervals (CIs). Least absolute shrinkage and selection operator across imputed datasets allowed identifying the candidate variable to include in the final multivariable model. The log-rank test was used to compare cumulative incidences of event in chronic and acute coronary syndrome subgroups. Finally, we conducted a sensitivity analysis, including only those participants who fulfilled the ARC-HBR criteria, and a per-protocol analysis, excluding participants who extended DAPT beyond 1 month. Supplementary material online, *Table S2* illustrates the list of major and minor ARC-HBR criteria and their respective definitions adapted to the current study database. A two-sided *P*-value of <0.05 was considered statistically significant. All statistical analyses were performed by using R 4.3.1.

## Results

### Clinical and procedural characteristics

After excluding 596 and 281 participants who received a BMS or more than 1 month of DAPT after PCI, the final study population included 766 participants, 323 originally enrolled in the SENIOR trial and 443 in the POEM trial (see Supplementary material online, *Figure S1*).

*Table 1* reports clinical characteristics at baseline. Participants had a mean age of 77.5 ± 8.2, and women were 31.9%. The ischemic and bleeding risk of the population can be inferred by the prevalence of chronic kidney disease (CKD, 37.7%), diabetes mellitus (34.8%), prior PCI (34.7%), peripheral artery disease (18.7%), prior stroke (6.9%), and atrial fibrillation (35.1%). The most common clinical presentation was chronic coronary syndromes (71.2%), followed by non-ST segment elevation myocardial infarction (13.4%), unstable angina (10.8%), and lastly, ST-segment elevation myocardial infarction (4.6%). Almost all the participants (99.6%) had at least one minor ARC-HBR criterion, and 609 (79.5%) fulfilled the ARC criteria for HBR (see Supplementary material online, *Table S3*). Age and oral anticoagulation were the most common minor and major ARC-HBR criteria, respectively (see Supplementary material online, *Figure S2*).

**Table 1:** Baseline clinical characteristics

	Patients = 766
Age (years)	77.5 ± 8.2
Female	244 (31.9)
BMI (kg/m^2^)	27.6 ± 5.1
Hypertension	620 (85.3)
Hypercholesterolemia	478 (66.3)
Current smoking	74 (10.8)
Family history	157 (24.5)
Diabetes mellitus	251 (34.8)
Diabetes mellitus on insulin therapy	83 (11.5)
Prior myocardial infarction	64 (27.9)
Prior PCI	230 (34.7)
Prior CABG	58 (8.7)
Prior stroke	46 (6.9)
Peripheral artery disease	122 (18.7)
Atrial fibrillation	233 (35.1)
Chronic kidney disease	229 (37.7)
Clinical presentation	
Chronic coronary syndrome	543 (71.2)
Unstable angina	82 (10.8)
NSTEMI	102 (13.4)
STEMI	35 (4.6)
Malignancy	87 (14.4)
Blood disorders	396 (65.5)
Liver disease	16 (2.7)
Haemoglobin (g/dL)	13 ± 1.9
Platelets (cells × 10^9^/L)	218 ± 71.9
Serum creatinine (mg/dL)	1.3 ± 1.1
Estimated glomerular filtration rate (mL/min/1.73 m^2^)	66.5 ± 22.5

Values are mean ± SD or number (%).

Chronic kidney disease was defined by an estimated glomerular filtration rate < 60 mL/min/1.73 m^2^.

BMI, body mass index; CABG, coronary artery bypass grafting; CAD, coronary artery disease; NSTEMI, non-ST-segment elevation myocardial infarction; PCI, percutaneous coronary intervention; STEMI, ST-segment elevation myocardial infarction.

*Table 2* reports the procedural characteristics. The left anterior descending artery was the most frequent target vessel being treated in 470 (44.5%) of the 1119 lesions. The mean number of lesions per patient was 1.4 ± 0.6. Complete revascularization and procedural success were achieved in 95.2% and 99.9% of the cases, respectively.

**Table 2:** Angiographic and procedural characteristics

	Patients = 766Lesions = 1119
Lesions/patient	1.4 ± 0.6
Target vessel^b^	
Left main	52 (4.9)
Left anterior descending	470 (44.5)
Left circumflex	242 (22.9)
Right coronary artery	292 (27.7)
Aspirin before PCI^a^	198 (25.8)
P2Y_12_ inhibitor loading before PCI^a^	366 (47.8)
Complete revascularization^a^	729 (95.2)
Procedural success^a^	765 (99.9)
Diameter stenosis (%)^b^	82.4 ± 12.5
Lesion length (mm)^b^	24.1 ± 16.6
Reference vessel diameter (mm)^b^	3.3 ± 3.4
De novo^b^	1041 (93.4)
Bifurcation^b^	192 (17.2)

Values are mean ± SD or number (%).

PCI, percutaneous coronary intervention.

^a^Per patient.

^b^Per lesion.

A total of 589 participants (92.2%) were discharged with clopidogrel as a P2Y12 inhibitor, while ticagrelor and prasugrel were prescribed in only 41 (6.4%) and 9 (1.4%) of them, respectively (see Supplementary material online, *Table S4*). More than 85% of participants dropped the P2Y12 inhibitor after the first month (see Supplementary material online, *Figure S3*), with a median duration of 31 (30–34) days. Following DAPT discontinuation, 94.4% of participants continued with aspirin, while 5.6% continued with P2Y12 inhibitor.

### Outcomes

**Figure 1:**
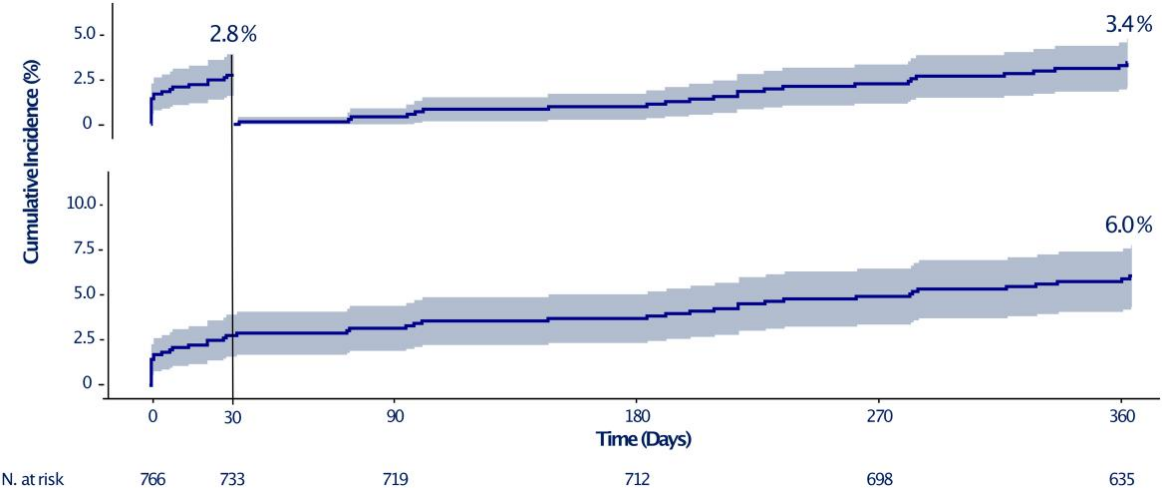
Incidence of the primary endpoint (MACCE) through 1 year of follow-up. Kaplan–Meier curves showing the cumulative incidence (95% CI) of the primary endpoint (MACCE, a composite of cardiovascular death, myocardial infarction, or stroke) at 1 year (lower) and landmark analysis at 1 month (upper).

One-year follow-up was completed in 93.9% of participants, with a median follow-up of 365.25 (365–365.25) days. *Table 3* reports the clinical outcomes at 1 year and the landmark analysis at 1 month. The primary endpoint occurred in 45 participants (6.0%; 95% CI, 4.3–7.7%) through 1 year of follow-up, with 21 participants (2.8%; 95% CI, 1.6–3.9%) affected during the first month and 24 participants (3.4%; 95% CI, 2.0–4.7%) thereafter (*Figure 1*). Among patients meeting the ARC-HBR criteria, the incidence of the primary endpoint was higher, with rates of 3.0% (95% CI, 1.6–4.3%) at 1 month and 7.0% (95% CI, 4.9–9.0%) at 1 year. The 1-year incidence of the individual components of the primary endpoint was 2.2% for cardiovascular death (95% CI, 0.36–2.50%), 3.1% for myocardial infarction (95% CI, 1.8–4.3%), and 1.2% for stroke (95% CI, 0.4–2.0%). Out of the five (two definite and three probable) cases of stent thrombosis (0.7%; 95% CI, 0.1–1.3%), two (0.3%; 95% CI, 0–0.6%) occurred in the first 30 days (at Days 1 and 14 under DAPT with aspirin and clopidogrel therapy) and three (0.4%; 95% CI, 0–0.9%) between 30 to 365 days after PCI (two at Days 210 and 219 during single antiplatelet therapy with aspirin, and one at Day 263 during single antithrombotic therapy with direct oral anticoagulant). BARC type 3–5 bleeding incidence was 1.1% (95% CI 0.3–1.8%) and 2.9% (95% CI, 1.6–4.1%) at 1 month and 1 year, respectively.

**Table 3:** Clinical outcomes at 0–30, 30–365, and 0–365 days

	0–30 days N. at risk = 766 N. (rate; 95% CI)	30–365 days N. at risk = 724–752 N. (rate; 95% CI)	0–365 daysN. at risk = 766 N. (rate; 95% CI)
MACCE	21 (2.8; 1.6–3.9)	24 (3.4; 2.0–4.7)	45 (6.0; 4.3–7.7)
NACCE	27 (3.5; 2.2–4.8)	31 (4.4; 2.9–5.9)	58 (7.8; 5.8–9.7)
All-cause death	4 (0.5; 0–1.0)	30 (4.2; 2.7–5.6)	34 (4.7; 3.1–6.2)
Cardiovascular death	2 (0.3; 0–0.6)	14 (1.9; 0.9–2.9)	16 (2.2; 1.1–3.2)
Non-cardiovascular death	2 (0.3; 0–0.6)	16 (2.3; 1.2–3.4)	18 (2.5; 1.4–3.7)
Myocardial infarction	17 (2.2; 1.2–3.3)	6 (0.8; 0.2–1.5)	23 (3.1; 1.8–4.3)
Stroke	2 (0.3; 0–0.6)	7 (1.0; 0.2–1.7)	9 (1.2; 0.4–2.0)
Definite/probable ST	2 (0.3; 0–0.6)	3 (0.4; 0.0–0.9)	5 (0.7; 0.1–1.3)
Definite ST	1 (0.1; 0–0.4)	1 (0.1; 0.0–0.4)	2 (0.3; 0.1–0.6)
Probable ST	1 (0.1; 0–0.4)	2 (0.3; 0.0–0.7)	3 (0.4; 0.1–0.9)
Target lesion revascularization	2 (0.3; 0–0.6)	8 (1.1; 0.3–1.9)	10 (1.4; 0.5–2.2)
Target vessel revascularization	3 (0.4; 0–0.8)	14 (2.0; 0.9–3.0)	17 (2.4; 1.2–3.5)
Bleeding BARC type 3–5	8 (1.1; 0.3–1.8)	13 (1.8; 0.8–2.8)	21 (2.9; 1.6–4.1)

BARC, Bleeding Academic Research Consortium; MACCE, major adverse cardiac and cerebrovascular event; NACCE, net adverse cardiac and cerebrovascular events; ST, stent thrombosis.

Percentages are incidences computed by the Kaplan–Meier method at the following time points: from 0 to 30 days, from 30 to 365 days, and from 0 to 365 days after the index procedure.

MACCE was a composite of cardiovascular death, myocardial infarction, or stroke.

NACCE was a composite of cardiovascular death, myocardial infarction, stroke, or bleeding BARC type 3–5.

The per-protocol analysis and the subgroup analysis according to clinical presentation are reported in Supplementary material online, *Tables S5* and *S6*, respectively.

**Figure 2:**
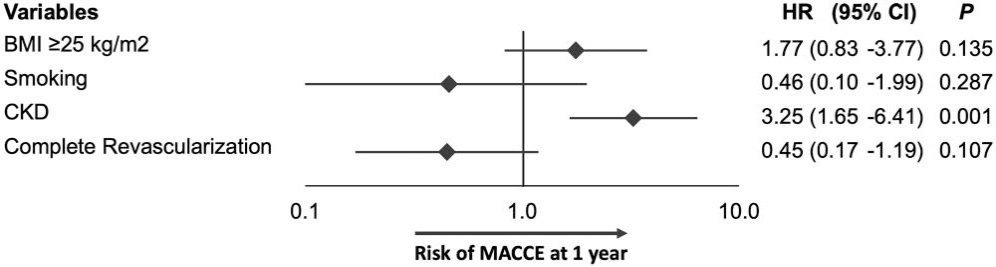
Predictors of the primary endpoint (MACCE) at 1 year. Forest plot for the predictors of the primary endpoint (MACCE, a composite of cardiovascular death, myocardial infarction, or stroke) at 1 year on multivariate analysis. Anaemia was defined as a haemoglobin level < 11 g/dL. CKD was defined as an estimated glomerular filtration rate < 60 mL/min/1.73 m^2^. Chronic kidney disease: CKD; HR: hazard ratio; BMI: body mass index; CI: confidence interval.

An estimated glomerular filtration rate < 60 mL/min/1.73 m^2^ was the only independent predictor of both the primary endpoint, i.e. MACCE (HR 3.25; 95%, CI 1.65–6.41; *P* = 0.001; *Figure 2*), and NACCE (HR 2.39; 95% CI, 1.33–4.29; *P* = 0.004; Supplementary material online, *Figure S4*). Anaemia was the only independent predictor of BARC type 3–5 bleeding (HR 3.39; 95% CI, 1.06–10.81, *P* = 0.041; Supplementary material online, *Figure S5*).

## Discussion

We provided comprehensive evidence on 1-month DAPT after Synergy EES stent by pooling 766 participants and 1119 lesions from the SENIOR and the POEM trials. In a population including approximately four out of five participants fulfilling the ARC-HBR criteria, the overall incidence of the primary and secondary endpoints was low. In particular, the rates of ischemic events occurring after the DAPT interruption (i.e. generally 1 month after the index PCI) compared favourably with those occurring within the first month (i.e. during DAPT). These results are consistent with available literature on other contemporary DES.^6,17–19^ Among our participants, those suffering from CKD were at higher risk for MACCE and NACCE, while those with anaemia were at higher risk of BARC type 3–5 bleeding after adjusting for confounders.

### High-bleeding risk patients

As of today, several definitions for the bleeding risk have been adopted in clinical trials. The POEM trial applied the same criteria of the LEADERS FREE and the ONYX ONE trial to select patients at HBR.^17,18^ In all of them, the most represented criterion was age ≥ 75 years, followed by oral anticoagulation, anaemia, or CKD. In the SENIOR trial, the patients were eligible if aged 75 years or older. The Academic Research Consortium aimed to standardize and reduce the heterogeneity in the bleeding risk definition among patients undergoing PCI.^20^ However, this consensus was reached only in 2019 and still requires prospective application in clinical trials. In the present study, 79.5% of patients fulfilled the ARC-HBR criteria, which is double the prevalence observed in real-world PCI cohorts.^3^ As discussed in the following paragraph, this premise is necessary to consider an early DAPT interruption.

### One-month DAPT after biodegradable-polymer EES

**Figure 3:**
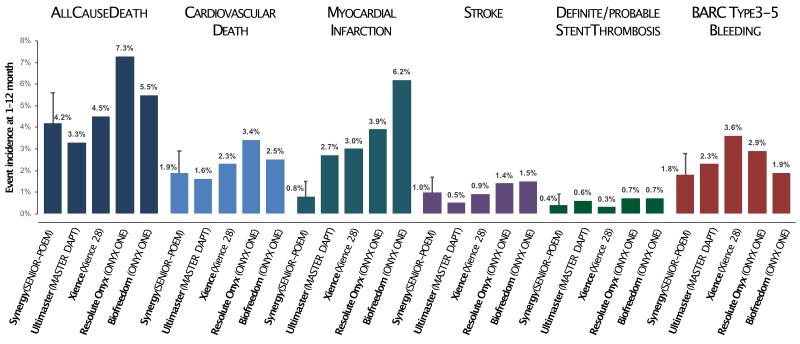
Findings in perspectives. Event rates observed between 1 and 12 months follow-up in contemporary DES trials treating HBR patients with a 1-month DAPT regimen following PCI. The Synergy biodegradable-polymer everolimus-eluting stent was used in the present study (i.e. POEM–SENIOR). The Ultimaster biodegradable-polymer sirolimus-eluting stent was used in the MASTER DAPT trial. The Xience durable-polymer everolimus-eluting stent was used in the XIENCE 28 trial. The Resolute Onyx durable-polymer zotarolimus-eluting stent and the BioFreedom polymer-free biolimus-eluting stent were used in the ONYX ONE trial. Event rates are Kaplan–Meier estimates, except for the XIENCE 28 reporting propensity score stratified mean rates. BARC: Bleeding Academic Research Consortium.

In the first period following PCI with stent implantation, the benefit of intensive antithrombotic therapy with DAPT generally outweighs the increased risk of bleeding. However, this advantage dissipates over time, favouring antithrombotic strategies that consider this trade-off. Based on the PRECISE-DAPT score, patients at HBR might benefit from an abbreviated DAPT duration of 3–6 months, while the others could receive a standard (12 months) or prolonged (>12 months) treatment without being exposed to significant bleeding liability.^4^ The MASTER DAPT suggested that DAPT duration could be further shortened to 1 month in HBR patients undergoing PCI with Ultimaster SES.^6,21^ Accordingly, most updated guidelines consider dropping one antiplatelet agent between aspirin and P2Y12 after 1 month in this selected group of patients.^22^ The main concern relates to the subsequent risk of ischemic events, such as myocardial infarction and stent thrombosis. Our study population exhibited characteristics associated with ischemic risk similar to those of an all-comers PCI cohort due to limited exclusion criteria (planned major surgery within a month, life expectancy less than 1 year, and cardiogenic shock as the PCI indication).^23,24^ The rates of ischemic and bleeding events occurring after the first month (i.e. the time point for intended DAPT interruption) were reassuring if compared with the rates occurring during the first month in the same population (*Figure 1*) and with those reported in the literature (*Figure 3*).^6,18,19^ Despite the absence of a comparator arm, the landmark analysis allowed for an internal comparison within the same population under similar conditions, except for the antithrombotic therapy. The incidence of all-cause death in this study was comparable to other studies (*Figure 3*). Such an outcome may reflect the baseline patient risk more than the treatment strategy itself. Conversely, the relatively low incidence of myocardial infarction and late stent thrombosis points to the safety of a short DAPT regimen following Synergy EES implantation. Specifically, the rate of late definite or probable stent thrombosis (i.e. between 1 and 12 months post-implantation) was 0.4% in our study compared to 0.3% in the XIENCE 28, 0.7% in the ONYX ONE, and 0.6% in the MASTER DAPT trial. Only the latter tested the non-inferiority of 1 month of DAPT compared to the continuation of therapy for at least two additional months in patients receiving Ultimaster SES. Still, these results suggest that this strategy might be applied to other biodegradable-polymer devices (i.e. the Synergy EES) and other contemporary DES (i.e. the Xience durable-polymer EES (Abbott, USA), the Resolute Onyx durable-polymer zotarolimus-ES (ZES, Medtronic, USA), and the BioFreedom polymer-free biolimus-ES (Biosensors International, Singapore).

The Synergy EES, similar to the Xience EES (81 μm), the Resolute Onyx ZES (81 μm), and the Ultimaster SES (80 μm), has very thin struts (74–81 μm) that reduce acute platelet aggregation associated with early ST and incomplete reendothelialization associated with late ST.^7,25^ The antiproliferative drug (i.e. everolimus) is completely released after 3 months, and the ultrathin and abluminal polymer is resorbed after an additional month.^8^ Of note, compared to a similar BMS, stent reendothelialization is not impacted by the presence of everolimus or the polymer, reaching a coverage rate of 87.5% (74.0–93.3%) above and 97.7% (96.9–98.0%) between the stent struts.^7^ In patients evaluated with optical coherence tomography, the intimal coverage was 94.5% ± 4.4% and 96.6% ± 2.7% after 3 and 6 months from Synergy EES implantation.^9^ In this period, intensive antithrombotic therapy can reduce the risk of ST proportional to this minor increase in reendothelialization. However, this may not be a significant advantage, but rather detrimental in patients at HBR, where the prognosis depends more on bleeding events.

We could not evaluate the impact of intravascular imaging to guide PCI and DAPT duration. Optical coherence tomography or intravascular ultrasound was used in about 5% of the POEM population. These rates align with European clinical practice according to a recent survey from the European Association of Percutaneous Cardiovascular Interventions.^26^ Despite evidence that intravascular imaging improves patient outcome,^27^ short DAPT appears safe, irrespective of the procedure's complexity or intravascular ultrasound (IVUS) use.^28^ In the IVUS-ACS trial presented at ACC 2024, there was no evidence of interaction between the type of guidance (IVUS vs. angiography alone) and DAPT duration (1 vs. 12 months). However, this analysis remains exploratory, and until more evidence becomes available, intravascular imaging is advisable, irrespective of the intended DAPT duration.

Finally, we explored if 1 month of DAPT was associated with signs of harm in particular subgroups of patients. No differences in the risk of adverse events were found between chronic and acute coronary syndrome subgroups. We identified only CKD as an independent predictor of the primary endpoint and NACE. Although CKD is a recognized bleeding risk factor, part of the ARC-HBR criteria,^20^ PRECISE-DAPT,^4^ or inclusion criteria of several HBR trials,^6,17,18^ it is actually included in most prediction models for both haemorrhagic and thrombotic events.^29–31^ As a result, patients with CKD still represent a challenging population where the optimal DAPT duration requires further evaluation.

### Study limitations

The present study should be interpreted in view of several limitations. First, our findings remain explorative because of the absence of a comparator arm, such as a different DAPT regimen or another DES. This lack of a control group limits our ability to draw definitive conclusions, and future randomized controlled trials with appropriate comparator arms are needed to validate these findings. Second, the two original studies had different designs. In more detail, SENIOR was a randomized trial comparing two different stents, while POEM was a single-arm registry enrolling participants after the procedure, which might explain the procedural success rate of 99.9%. This might have resulted in the inclusion of lower-risk patients in the POEM trial. As a result, we cannot extrapolate our findings to patients with suboptimal PCI results. Nevertheless, choosing the optimal DAPT duration is a dynamic process that should not be decided upfront the procedure, but must consider every new piece of information, such as stent apposition or any ischemic or bleeding complications during or after the procedure. Third, we acknowledge the increased risk of a type II error because of the small sample size. Fourth, the follow-up loss at 1 year exceeded 6%, which is high for a monitored clinical trial. This ascertainment bias should be acknowledged when interpreting our results. Fifth, the bleeding risk definition used in our study does not fully align with the ARC-HBR criteria, which identify patients at an even higher risk of adverse events. This discrepancy represents a limitation of our study, shared with other HBR trials published to date. Therefore, dedicated future prospective studies are necessary to address this issue comprehensively. Finally, the representation of patients with acute coronary syndrome from the SENIOR was limited because most of them received 6 months of DAPT and were excluded from the present analysis. However, our subgroup analysis by clinical presentation did not raise concern regarding the application of our strategy in HBR patients following an acute coronary syndrome.

## Supplementary Material

oeae068_Supplementary_Data

## Data Availability

The data underlying this article will be shared upon reasonable request to the corresponding author.

## Lead author biography

Carlo Andrea Pivato was born in Padova, Italy. He studied medicine at Vita-Salute San Raffaele University in Milan, graduating cum laude. He completed his residency in cardiology at Humanitas University. He then undertook a research fellowship at the Icahn School of Medicine at Mount Sinai in New York. He is currently a PhD researcher at Humanitas University and a cardiologist at Humanitas Research Hospital.

## Conclusion

HBR patients undergoing PCI with a biodegradable-polymer EES had few ischemic and bleeding events when given a DAPT of 1 month. One-month DAPT after biodegradable-polymer EES implantation can be a safe and effective strategy for patients with high bleeding risk, but further comparative studies are needed to validate these findings.
