# Supplementary material for: One-month DAPT after biodegradable-polymer everolimus-eluting stent implantation in patients at high-bleeding risk: an individual patient data pooled analysis of the SENIOR and POEM trials
Source: Eur Heart J Open. 2024 Aug 6;4(4):oeae068. doi: 10.1093/ehjopen/oeae068 (PMC11339713; doi:10.1093/ehjopen/oeae068)
Supplement: oeae068_Supplementary_Data [file oeae068_supplementary_data.docx]

**SUPPLEMENTAL MATERIAL**

**One-month DAPT after biodegradable-polymer everolimus-eluting stent implantation in patients at high-bleeding risk: an individual patient data pooled analysis of the SENIOR and POEM trials**

**Table of Contents**

**Supplementary Table 1.** Summary of the SENIOR and POEM trials.

**Supplementary Table 2.** Study definitions for major and minor ARC-HBR criteria compared with the original consensus document.

**Supplementary Table 3.** ARC-HBR score distribution in the total population.

**Supplementary Table 4.** P2Y_12_ Inhibitor and oral anticoagulation at discharge.

**Supplementary Table 5.** Intention-to-treat and per-protocol analyses evaluating outcomes at 1 year follow-up.

**Supplementary Table 6.** One-year clinical outcomes according to chronic or acute coronary syndrome.

**Supplementary Figure 1.** Study flowchart.

**Supplementary Figure 2.** Prevalence of the ARC-HBR criteria in the total population (N=766 patients).

**Supplementary Figure 3.** Duration of P2Y12 inhibitor.

**Supplementary Figure 4.** Predictors of NACCE at 1 year.

**Supplementary Figure 5.** Predictors of BARC type 3-5 bleeding at 1 year.

**Supplementary Table 1.** Summary of the SENIOR and POEM trials.

|  | **SENIOR** | **POEM** |
| --- | --- | --- |
| **Year of publication** | 2018 | 2022 |
| **N. of patients** | 1200 | 443 |
| **Study design** | Randomized controlled trial | Prospective single arm trial |
| **Aim** | Synergy EES vs BMS | Synergy EES vs OPC |
| **DAPT regimen** | 1 month for CCS, 6 months for ACS | 1 month |
| **Main inclusion criteria** | - - Age ≥75 years | ≥1 HBR criteria among:   - - Age ≥75 years   - Need for oral anticoagulation   - Hemoglobin <11 g/L   - Blood transfusion < 4 weeks   - Platelets <100’000/ mL   - Hospitalization for bleeding <1 year   - Stroke <1 year   - Prior intracerebral hemorrhage   - Severe chronic liver disease   - Creatinine clearance<40 ml/min   - Cancer <3 years   - Planned major surgery <1 year   - Need for glucocorticoids/NSAIDs   - Non-adherence to >30 days DAPT |
| **Main exclusion criteria** | - - Planned major surgery <1 month   - Life expectancy <1 year | - - Cardiogenic shock |
| **Primary endpoint** | All-cause death, MI, stroke, or target lesion revascularization at 1 year | Cardiac death, MI, or definite/probable ST at 1 year |
| **Endpoint adjudication** | Independent Clinical Event Committee | Independent Clinical Event Committee |
| **MI definition** | III Universal Definition [13] | III Universal Definition [13] |
| **ST definition** | ARC [14] | ARC [14] |
| **Bleeding definition** | BARC [15] | BARC [15] |

ACS: acute coronary syndrome; ARC: Academic Research Consortium; BARC: Bleeding Academic Research Consortium; BMS: bare metal stent; CCS: chronic coronary syndrome; DAPT: dual antiplatelet therapy; EES: everolimus-eluting stent; HBR: high bleeding risk; MI: myocardial infarction; NSAID: non-steroidal anti-inflammatory drug; OPC: objective performance criterion; ST: stent thrombosis.

**Supplementary Table 2.** Study definitions for major and minor ARC-HBR criteria compared with the original consensus document.

| ARC-HBR criteria | Original definition | ADAPTED definition |
| --- | --- | --- |
| **Major criteria** |  |  |
| Oral anticoagulation | Anticipated use of long-term oral anticoagulation | No difference |
| CKD, major | eGFR <30 mL/min | No difference |
| Anemia, major | Hemoglobin <11 g/dL | No difference |
| Prior major bleeding | Spontaneous bleeding requiring hospitalization or transfusion in the past 6 months or at any time, if recurrent | Not available |
| Thrombocytopenia | Platelet count <100 x 10^9/L | No difference |
|  | Chronic bleeding diathesis | Not available |
| Severe chronic liver disease | Liver cirrhosis with portal hypertension | Severe chronic liver disease |
| Cancer | Within the past 12 months (excluding non-melanoma skin cancer) | Within the past 36 months (excluding non-melanoma skin cancer) |
| Previous ICH | Spontaneous (at any time) | Not available |
|  | Previous traumatic ICH within the past 12 months | Not available |
|  | Presence of a bAVM | Not available |
| Prior stroke | Moderate or severe ischemic stroke within the past 6 months | Any prior stroke |
| Nondeferrable major surgery | Nondeferrable major surgery on DAPT | Not available |
|  | Recent major surgery or major trauma within 30 days before PCI | Not available |
| **Minor criteria** |  |  |
| Age≥75 years | Age ≥75 years | No difference |
| CKD, minor | eGFR 30–59 mL/min | No difference |
| Anemia, minor | Hemoglobin 11–12.9 g/dL for men and 11–11.9 g/dL for women | No difference |
|  | Prior major bleeding not meeting major criterion | Not available |
| Steroids or NSAIDS use | Long-term use of oral NSAIDs or steroids | Not available |
|  | Any ischemic stroke at any time not meeting the major criterion | Not available |

bAVM: brain arteriovenous malformation; CKD: chronic kidney disease; DAPT: dual antiplatelet therapy; eGFR: estimated glomerular filtration rate; ICH: intracranial hemorrhage; NSAIDs: non-steroidal anti-inflammatory drugs; PCI: percutaneous coronary intervention.

**Supplementary Table 3.** ARC-HBR score distribution in the total population.

| **ARC-HBR score** | **PATIENTS**  **(N=766)** |
| --- | --- |
| 0 | 3 (0.4) |
| 0.5 | 154 (20.1) |
| 1 | 123 (16.1) |
| 1.5 | 158 (20.6) |
| 2 | 131 (17.1) |
| 2.5 | 112 (14.6) |
| 3 | 41 (5.4) |
| 3.5 | 24 (3.1) |
| 4 | 9 (1.2) |
| 4.5 | 6 (0.8) |
| 5 | 4 (0.5) |
| 5.5 | 1 (0.1) |

Values are n (%).

The score is calculated as follows: each minor criterion is assigned a value of 0.5, and each major criterion is assigned a value 1. The sum of these values is summed in the total score. Patients are deemed to fulfill the ARC-HBR criteria if their score is ≥1.

**Supplementary Table 4.** P2Y_12_ Inhibitor and oral anticoagulation at discharge.

|  | **PATIENTS**  **(N=766)** |
| --- | --- |
| P2Y_12_ Inhibitor |  |
| Clopidogrel | 589 (92.2) |
| Prasugrel | 9 (1.4) |
| Ticagrelor | 41 (6.4) |
| Days duration before interruption | 31 (30 - 34) |
| Oral anticoagulation |  |
| Apixaban | 41 (23.8) |
| Dabigatran | 27 (15.7) |
| Edoxaban | 17 (9.9) |
| Rivaroxaban | 41 (23.8) |
| Vitamin K Antagonist | 45 (26.2) |

Values are median (interquartile range) or n (%).

**Supplementary Table 5.** Intention-to-treat and per-protocol analyses evaluating outcomes at 1 year follow-up.

|  | Intention-to-treat  N. at risk=766  N. (RATE; 95%CI) | PEr-protocol  N. at risk=543  N. (RATE; 95%CI) |
| --- | --- | --- |
| MACCE | 45 (6.0; 4.3-7.7) | 29 (5.47;3.51-7.39) |
| NACCE | 58 (7.8; 5.8-9.7) | 37 (6.94;4.76-9.07) |
| All-cause death | 34 (4.7; 3.1-6.2) | 24 (4.64;2.8-6.44) |
| Cardiovascular death | 16 (2.2; 1.1-3.2) | 12 (2.3;1-3.58) |
| Non-cardiovascular death | 18 (2.5; 1.4-3.7) | 12 (2.39;1.04-3.73) |
| Myocardial infarction | 23 (3.1; 1.8-4.3) | 15 (2.79;1.39-4.17) |
| Stroke | 9 (1.2; 0.4-2.0) | 5 (0.94;0.12-1.76) |
| Definite/probable ST | 5 (0.7; 0.1-1.3) | 2 (0.38;0-0.9) |
| Definite ST | 2 (0.3; 0.1-0.6) | 1 (0.19;0-0.55) |
| Probable ST | 3 (0.4; 0.1-0.9) | 1 (0.19;0-0.57) |
| Target lesion revascularization | 10 (1.4; 0.5-2.2) | 7 (1.37;0.35-2.38) |
| Target vessel revascularization | 17 (2.4; 1.2-3.5) | 12 (2.31;1.01-3.6) |
| Bleeding BARC type 3-5 | 21 (2.9; 1.6-4.1) | 14 (2.7;1.29-4.09) |

Percentages are incidences computed by the Kaplan–Meier method.

BARC: Bleeding Academic Research Consortium; MACCE: major adverse cardiac and cerebrovascular event; NACCE: net adverse cardiac and cerebrovascular events; ST: stent thrombosis.

**Supplementary Table 6.** One-year clinical outcomes according to chronic or acute coronary syndrome.

|  | CCS  N. at risk=543  N. (RATE; 95%CI) | ACS  N. at risk=219  N. (RATE; 95%CI) | P-value |
| --- | --- | --- | --- |
| MACCE | 31 (5.84; 3.82-7.82) | 14 (6.65; 3.22-9.97) | 0.683 |
| NACCE | 40 (7.55; 5.27-9.78) | 18 (8.41; 4.61-12.05) | 0.646 |
| All-cause death | 23 (4.44; 2.65-6.21) | 11 (5.31; 2.2-8.32) | 0.609 |
| Cardiovascular death | 11 (2.09; 0.86-3.31) | 5 (2.44; 0.3-4.54) | 0.797 |
| Non-cardiovascular death | 12 (2.4; 1.05-3.74) | 6 (2.93; 0.59-5.22) | 0.646 |
| Myocardial infarction | 15 (2.81; 1.4-4.2) | 8 (3.73; 1.16-6.23) | 0.499 |
| Stroke | 8 (1.51; 0.47-2.54) | 1 (0.51; 0-1.5) | 0.250 |
| Definite/probable ST | 3 (0.57; 0-1.21) | 2 (0.95; 0-2.25) | 0.565 |
| Definite ST | 1 (0.19; 0-0.55) | 1 (0.49; 0-1.44) | 0.500 |
| Probable ST | 2 (0.38; 0-0.92) | 1 (0.46; 0-1.36) | 0.848 |
| Target lesion revascularization | 7 (1.38; 0.35-2.38) | 3 (1.47; 0-3.1) | 0.906 |
| Target vessel revascularization | 10 (1.95; 0.74-3.14) | 7 (3.46; 0.9-5.96) | 0.237 |
| Bleeding BARC type 3-5 | 13 (2.48; 1.14-3.81) | 8 (3.85; 1.19-6.44) | 0.325 |

The cumulative incidences of each end point in CCS and ACS subgroups were estimated using the Kaplan−Meier method and were compared using the log-rank test.

ACS: acute coronary syndrome; BARC: Bleeding Academic Research Consortium; CCS: chronic coronary syndrome; MACCE: major adverse cardiac and cerebrovascular event; NACCE: net adverse cardiac and cerebrovascular events; ST: stent thrombosis.

**Supplementary Figure 1. Study flowchart.** The figure shows how the final study population was selected.

* As intended at discharge.

BMS: bare metal stent; DAPT: dual antiplatelet therapy; EES: everolimus-eluting stent.

**Supplementary Figure 2. Prevalence of the ARC-HBR criteria in the total population (N=766 patients).**

Anemia, minor: hemoglobin 11-12.9 g/dL in men, 11-11.9 g/dL in women.

Anemia, major: hemoglobin < 11 g/dL.

CKD, minor: estimated glomerular filtration rate 30 – 60 mL/min/1.73 m^2^.

CKD, major: estimated glomerular filtration rate < 30 mL/min/1.73 m^2^.

*If any information was missing, we assumed the criterion was unmet.

**Supplementary Figure 3. Duration of P2Y12 inhibitor.**

**Supplementary Figure 4. Predictors of NACCE at 1 year.**

Forrest plot for the predictors of NACCE (a composite of cardiovascular death, myocardial infarction, stroke, or bleeding BARC type 3-5) at 1 year on multivariate analysis. Anemia was defined as a hemoglobin level<11 g/dL. Anemia was defined as a hemoglobin level<11 g/dL. CKD was defined as an estimated glomerular filtration rate < 60 mL/min/1.73 m2. Chronic kidney disease: CKD; HR: hazard ratio; CI: confidence interval.

**Supplementary Figure 5. Predictors of BARC type 3-5 bleeding at 1 year.**

Forrest plot for the predictors of BARC 3-5 bleeding at 1 year on multivariate analysis. Anemia was defined as a hemoglobin level<11 g/dL. CKD was defined as an estimated glomerular filtration rate < 60 mL/min/1.73 m2. Chronic kidney disease: CKD; HR: hazard ratio; CI: confidence interval.
